# State-of-the-Art Polymer Science and Technology in Italy

**DOI:** 10.3390/polym12081721

**Published:** 2020-07-31

**Authors:** Ignazio Blanco, Roberto Pantani, Antonio Pizzi, Andrea Sorrentino

**Affiliations:** 1 Department of Civil Engineering and Architecture and INSTM UdR, University of Catania, V.le A. Doria 6, 95125 Catania, Italy; 2 Industrial Engineering Department, University of Salerno, I-84084 Fisciano (Salerno), Italy; 3 LERMAB, Laboratoire d’Etude et de Recherche sur le MAteriau Bois, Université de Lorraine, 27 rue Philippe Seguin, CS60036, 88021 Epinal, France; 4 Institute of Polymers, Composites and Biomaterials (IPCB), National Research Council of Italy (CNR), Via Previati 1/C, 23900 Lecco, Italy

The history of polymers in Italy certainly begins with Giulio Natta and the discovery of isotactic polypropylene [1,2], culminated in the delivery to Prof. Natta of the Nobel Prize for chemistry in 1963. Before those years, thanks to the development of the hydrocarbons and petrochemical sector by Montecatini (General Society for the Mining and Chemical Industry), the foundations for the development of macromolecular chemistry in Italy had been laid, but the real expansion occurred between 1958 and 1967 with a sector growth of 30% on an annual basis [3]. The research of Natta and his collaborators, on metal-organic catalysis applied to the polymerization reactions of -olefins and other unsaturated monomers, promoted the growth and consolidation, in Italy, of a network of industrial centers and academic institutes operating in the polymer science and technology sector, creating international skills. Since that period until today, Italy has kept pace with other countries and has certainly contributed to the development of the polymeric materials sector. Today, polymer science and technology in Italy represents a long-lasting and interdisciplinary field in which chemistry, physics, and engineering mix together to produce studies, which are considered among the best in the world. Several research groups coming from very different fields often collaborate in the design of the material, of the part, and of the processing technology to obtain innovative products with outstanding, new, and smart properties. Examples of the contributions of Italian research in the field are spread out in the top Journals and Conferences throughout the world. This Special Issue, which consists of 22 articles, including two review articles, aims at collecting an overview of Polymer Science and Technology in Italy, hoping that it can portray the state of the Polymeric Research in Italy and give the rest of the world a bright image of what is carried out in the field in our country.

In the molecular imprinting technique, the use of preformed oligomers instead of functional monomers increases the stability of the non-covalent interactions with the template molecule, providing a sharp gain in terms of binding properties for the resulting imprinted polymer. Based on this theory, Baggiani and co-workers assumed that the delayed addition of template molecules to a polymerization mixture enhances the binding properties of the resulting polymer, concluding that the delayed addition approach could be useful in prepare imprinted polymers with higher binding affinity and increased binding selectivity in cases of difficult imprinting polymerization [4].

Catauro et al. investigated the effectiveness of nanocomposites (composed of ultra-high molecular weight polyethylene (UHMWPE) mixed with carbon nano-filler (CNF) and medical grade paraffin oil (PO), from the biological point of view. They carried out wear measurements without (air) and with lubricant (distilled water, natural, and artificial lubricant) and evaluated the antibacterial activity and cytotoxicity. Their results highlighted that the presence of CNF is important in the nanocomposite formulation because it reduces the wear rate and prevents oxidative degradation during its processing [5].

Pucci et al. investigated the functionalization of polyketone 30 (PK30) with glycyl-glycine (Gly-Gly) via the Paal–Knorr reaction with the aim of homogenously dispersing two types of reduced graphene oxide (rGO, i.e., lrGO and hrGO, the former characterized by a lower degree of reduction in comparison to the latter) by non-covalent interactions. All the composites showed a typical semiconductive behavior and stable electrical response after several heating/cooling cycles from 30 to 115 °C, demonstrating their usability as an ON–OFF temperature sensor and as sensing material in soft robotics applications [6].

Malucelli et al. evaluated the influence of carbonaceous materials, such as biochar (BC) and/or multiwalled carbon nanotubes (MWCNTs), in the rheological and thermal properties of UV-LED curable coatings. They reported on the synthesis and characterization of carbon-reinforced films containing nanometric (MWCNTs) and micrometric (BC) carbon-based materials. They analyzed the rheological properties of the UV-LED curable dispersions, as well as the thermal and optical properties of the resulting films, establishing some correlations between filler dispersion/loading with the main observed properties [7].

Leporini and co-workers, by the means of molecular dynamics simulations, studied two melts of polymer chains with different bond length, resulting in rather different strength of the Johari-Goldstein (JG) relaxation. They found that, even if changing the bond length alters both the strength and the relaxation time of the JG relaxation, it leaves unaffected the correlation between the vibrational dynamics and the primary relaxation [8].

Film blowing technique was used to prepare polymer films based on biodegradable polymers, polyethylene (PE) and modified PE with oxo-degradable additive by La Mantia et al. [9]. The Sicilian researchers investigated mechanical properties, soil burial degradation and surface wettability of the obtained polymers, before and after UV irradiation. They observed that UV irradiation increased surface wettability and encouraged the disintegration in soil of all the samples, due to molar mass reduction and the production of hydrophilic end groups by photo-oxidation, thus increasing surface erosion and weight loss.

Tondi and co-workers produced high homogeneity tannin foams suitable for industrial upscaling. Foams catalyzed with nitric acid showed similar physical properties and more phenolic character, which favors the absorption of ionic pollutants. Conversely, the foams blown with aliphatic solvents and surfactants presented smaller pores, and higher mechanical and insulating properties, without affecting the chemical properties or the heating value. The combined foam produced with nitric acid as a catalyst and petroleum ether as a blowing agent result in sulfur-free and small cell material with overall improved features [10].

Gorrasi et al. studied the effect of ionic liquid (IL) in improving the dispersion of a Zn/Al layered double hydroxides (LDHs) hosting carbon nanotubes (80% of CNTs) into a commercial biodegradable highly amorphous vinyl alcohol polymer [11]. The analysis of thermal, mechanical and electrical properties of the composites, resulted improved compared to the unfilled material, allowed to hypothesize a good dispersion of the LDH-CNTs lamellar filler into the polymer matrix-assisted by the ionic liquid. This was demonstrated comparing electrical conductivity of composite at 5% of LDH-CNTs in the presence and in the absence of IL.

The monitoring of the properties’ development of different hybrid epoxy formulations, during low temperature curing and aging, was carried out by Frigione and co-workers [12]. The durability of the produced hybrids was probed in comparison to the corresponding neat epoxies by glass transition temperature (*T*_g_) measurements. Flexural mechanical properties after exposure to different levels of humidity and immersion in water and at temperatures slightly higher than the local ambient temperature were also evaluated, in order to simulate the conditions encountered during summer seasons in very humid environments. Their epoxy-based systems displayed significant advantages over the conventional epoxy resins used as structural adhesives or as matrices for fiber reinforced composites in terms of higher *T*_g_, better mechanical properties and enhanced durability in aqueous environments.

Piras and co-workers investigated the potential application of ammonium–chitosan grafted with 2-methyl-β-cyclodextrin (N^+^-rCh-MCD) as the functional macromolecular complexing agent for the oral administration of the neuropeptide dalargin (DAL). The results of NMR characterization, UV and fluorescence techniques, as well as biological in vitro assessments, indicated that N^+^-rCh-MCD forms water-soluble complexes with DAL, with a prevalent involvement of Tyr or Phe over Leu and Ala residues. The association constant of DAL with the polymeric derivative is one order of magnitude higher than that with the pristine cyclodextrin (K_a_: 2600 M^−1^ and 120 M^−1^, respectively). Additionally, N^+^-rCh-MCD shields DAL from enzymatic degradation in gastrointestinal in vitro models with a three-fold time delay, suggesting a future pharmaceutical exploitation of the polymeric derivative [13].

Aiming at understanding the relationship between the properties of the plastic objects and the process conduction, and thus the evaluation of process parameters, such as the mold temperature, Speranza et al. placed a thin electrical heater below the cavity surface in order to obtain rapid and localized surface heating/cooling cycles during the injection molding process [14]. The modulation of the cavity temperature was found able to control the distribution of relevant morphological characteristics, thus, properties along the sample thickness. The crystalline degree slightly increased with the cavity temperature, and this induced an increase in the elastic modulus when high temperatures were adopted for the cavity surface. The cavity temperature strongly influenced the orientation distribution that, on its turn, determined the highest values of the elastic modulus found in the shear layer. In particular, they observed that, if the macromolecules spent adequate time at temperatures close to the crystallization temperature, it was possible achieving high levels of structuring and, thus, high values of elastic modulus.

With the purpose to fulfill higher standard prerequisites and properties in dental restorative materials, such as high modulus, high hardness, and chemically inertness, Tammaro et al. developed novel composites with multiple biofunctions [15]. They prepared light-cured Bis-GMA/TEGDMA dental resin (RK)/layered double hydroxide intercalated with fluoride ions (LDH-F)/calcium bentonite (Bt) hybrid composites, recording an improvement of the mechanical properties with respect to the pristine resin. Furthermore, they observed, for the synthetized composites, antibacterial and antibiofilm effects against three bacterial strains isolated from healthy volunteers’ saliva (*Streptococcus* spp., *Bacteroides fragilis*, and *Staphylococcus epidermidis*) without affecting its ability to induce dental pulp stem cells differentiation into odontoblast-like cells, thus making these materials a promising strategy in preventive and restorative dentistry.

De Marco and Franco worked at the design and production of composite microparticles with a prolonged drug release for oral delivery. The supercritical antisolvent (SAS) process was used to coprecipitate Eudragit L100-55 (EUD) with diclofenac (DICLO) and theophylline (THEOP), obtaining well-defined spherical microspheres with a mean diameter ranging from 3.75 and 5.93 µm. After a wide characterization (scanning electron microscopy, differential scanning calorimetry, X-ray microanalysis, FT-IR, and UV–vis spectroscopy), they carried out dissolution studies, showing the potential of EUD to prolong the drug release, significantly, up to a few days [16].

In order to solve dark aspects related to the Polyfurfuryl alcohol (PFA) molecular structure derived from the polymerization in acid environment, Tondi et al. carried out a wide spectroscopic investigation [17]. Thanks to a multi-technique approach, involving Solid-State ^13^C-NMR, Attenuated Total Reflectance (ATR), Fourier Transform Infrared (FTIR) spectroscopy, and UV-resonant Raman spectroscopy at different excitation wavelengths, using both an UV laser source and UV synchrotron radiation and simulations with first-principles and semi-empiric methods to evaluate their matching with experimental ones, they concluded that, in addition to the major linear unconjugated polymerization, the PFA structure consists of Diels-Alder rearrangements occurring after the opening of some furanic units, while the terminal moieties of the chain involves γ-lactone arrangements. The occurrence of head-head methylene ether bridges and free hydroxyl groups (from unreacted furfuryl alcohol, FA, or terminal chains) could be excluded, while the conjugated systems could be considered rather limited.

In their work, Roppolo and colleagues presented new 3D printable materials based on the introduction of different commercially available ionic liquids (ILs) in the starting formulations. They evaluated the influence of these additives on the printability of such formulations through light-induced 3D printing (digital light processing—DLP), as well as investigating the effect of ionic liquids with polymerizable groups. The physical chemical properties of such materials were compared, focusing on the permeability towards CO_2_ of the different ILs present in the formulations, showing the possibility of 3D printing high complexity structures, which could be the base of new high complexity filters for a more efficient CO_2_ capture [18].

Lazzara et al. have developed active, healable, and safely dissolvable alginate-pectin-based biocomposites for possible applications in food packaging [19]. After morphological, mechanical, thermal tests, they observed properties in the range of commercially available packaging films. Furthermore, the prepared biocomposites exhibited higher hydrophobicity properties and antibacterial properties against different bacteria, making them ideal for utilization as packaging material.

Continuing their study on biodegradable polymers, La Mantia and co-workers prepared irrigation tubes by extruding polylactide/poly (butyleneadipate-co-butyleneterephthalate) (PLA/PBAT) blend (Bio-Flex^®^) and Mater-Bi^®^. After rheological and mechanical characterization, the obtained pipes were subjected to photoaging with continued exposure to UV radiation up to 22 days together with a soil burial degradation test at 30 °C and 50 °C for up to 70 days. They observed that the degradation rate of irrigation tube samples based on Mater-Bi^®^ was higher at 30 °C and was stimulated after 14 days of UV irradiation, thus concluding that higher temperatures or UV aging encouraged the disintegration in soil of Bio-Flex^®^-based irrigation tubes [20].

Pizzi and co-workers studied the reaction among Soy protein isolate (SPI) and insoluble soy flour polymeric carbohydrates with sodium periodate for the specific oxidation of vicinal –OH groups aiming at investigate the reactions involved in this approach to soy flour adhesives. They observed the generation of carbohydrate oligomer fractions presenting one, two or multiple aldehyde groups. The reaction of periodate with soy protein isolate has been shown to generate some aldehydes too, whilst when the mix of SPI and soy insoluble carbohydrates is treated with periodate, the majority of the observed aldehyde carrying species appear to be higher molecular weight carbohydrate oligomer fractions [21].

Two series of novel dumbbell-shaped polyhedral oligomeric silsesquioxanes (POSSs), fully functionalized with phenyl groups at the corner of the silicon cages, were synthesized by Blanco et al. to prepare, by the means of in situ polymerization, polystyrene (PS) nanocomposites. Thermal and morphological properties were evaluated and compared among the nanocomposites obtained using the two different series of dumbbell-shaped POSSs and with the net PS showing much higher values when compared with those of neat PS, and highlighting significant differences when an aliphatic or aromatic bridge was used to link the silicon cages [22].

A new formulation based on Diglycidyl ether of bisphenol A (DGEBA) mixed with Diethyltoluene diamine (DETDA) and with a commercial daylight photocurable resin was prepared by Tosto et al. to obtain printable materials in a liquid crystal display (LCD) 3D printer. After the calorimetric, dynamic mechanical and rheology testing, the authors carried out 3D printing trials, thus determining the best printing conditions [23].

In the first review of this Special Issue, Trotta and co-workers gave an overview of cyclodextrin-based nanosponges research, focusing on the origin and key points of the historical development in the last 50 years, progressing from relatively simple crosslinked networks in the 1960s to today’s multifunctional polymers. They explored the historical evolution of nanosponges in order to understand their role today, and imagine their future [24].

Another review by Siracusa et al. offered a rundown of life-cycle assessment (LCA) studies on polymers, used in the most important production and commercial sectors, carried out in the last few years by Italians researchers in order to remark, in the role of researchers who have made polymers and their derivatives their main research object, that a sustainable use of polymeric materials is not only possible but is, above all, necessary [25].
